# Complete Sequencing and Pan-Genomic Analysis of *Lactobacillus delbrueckii* subsp. *bulgaricus* Reveal Its Genetic Basis for Industrial Yogurt Production

**DOI:** 10.1371/journal.pone.0015964

**Published:** 2011-01-17

**Authors:** Pei Hao, Huajun Zheng, Yao Yu, Guohui Ding, Wenyi Gu, Shuting Chen, Zhonghao Yu, Shuangxi Ren, Munehiro Oda, Tomonobu Konno, Shengyue Wang, Xuan Li, Zai-Si Ji, Guoping Zhao

**Affiliations:** 1 Key Laboratory of Systems Biology/Key Laboratory of Synthetic Biology, Shanghai Institutes for Biological Sciences, Chinese Academy of Sciences, Shanghai, China; 2 Shanghai-MOST Key Laboratory of Health and Disease Genomics, Chinese National Human Genome Center at Shanghai, Shanghai, China; 3 Division of Research and Development, Meiji Dairies Corporation, Odawara, Japan; 4 Shanghai Centre for Bioinformation Technology, Shanghai, China; 5 School of Life Science, Fudan University, Shanghai, China; 6 Graduate School of the Chinese Academy of Sciences, Shanghai, China; 7 Department of Microbiology and Li Ka Shing Institute of Health Sciences, The Chinese University of Hong Kong, Prince of Wales Hospital, Shatin, New Territories, Hong Kong SAR, China; University of Hyderabad, India

## Abstract

*Lactobacillus delbrueckii* subsp. *bulgaricus* (*Lb. bulgaricus*) is an important species of Lactic Acid Bacteria (LAB) used for cheese and yogurt fermentation. The genome of *Lb. bulgaricus* 2038, an industrial strain mainly used for yogurt production, was completely sequenced and compared against the other two ATCC collection strains of the same subspecies. Specific physiological properties of strain 2038, such as lysine biosynthesis, formate production, aspartate-related carbon-skeleton intermediate metabolism, unique EPS synthesis and efficient DNA restriction/modification systems, are all different from those of the collection strains that might benefit the industrial production of yogurt. Other common features shared by *Lb. bulgaricus* strains, such as efficient protocooperation with *Streptococcus thermophilus* and lactate production as well as well-equipped stress tolerance mechanisms may account for it being selected originally for yogurt fermentation industry. Multiple lines of evidence suggested that *Lb. bulgaricus* 2038 was genetically closer to the common ancestor of the subspecies than the other two sequenced collection strains, probably due to a strict industrial maintenance process for strain 2038 that might have halted its genome decay and sustained a gene network suitable for large scale yogurt production.

## Introduction

Lactic Acid Bacteria (LAB), a heterogeneous group of Gram-positive bacteria, are extensively present in nature, and widely used for fermenting a variety of raw food and feeds primarily to produce lactic acid [1], [2]. The *Lactobacillus delbrueckii* subsp. *bulgaricus* (*Lb. bulgaricus*, hereafter), one of the three subspecies of *Lb. delbrueckii*, is a facultatively anaerobic, non-motile and non-spore-forming, rod-shaped member of LAB [3]. *Lb. bulgaricus* acts synergistically with *Streptococcus thermophilus* as “thermophilic” starter cultures in the manufacturing of yogurt. At an optimal temperature of approximately 42°C, these cultures grow fast and acidify quickly with desired organoleptic properties.

Recently, substantial progress has been achieved in genomic sequencing of LAB, including two collection strains of *Lb. bulgaricus*, ATCC 11842 [4] and ATCC BAA-365 [5] and some novel features of *Lb. bulgaricus* were identified [4]. Comparative genomic analyses revealed that the *Lb. bulgaricus* genome has undergone a rapid reductive evolution as gene loss and metabolic simplification, known to be the central trend of evolving LABs [5]. In addition, genomic analysis implicated the physiological basis for proto-cooperation between *Lb. bulgaricus* and *S. thermophilus* [4].

In this paper, we present the complete genomic sequence of *Lb. bulgaricus* 2038, an industrial strain used by Meiji Dairies Corporation originally isolated from Bulgaria. Comparative genomic analysis against two other collection strains of the same subspecies revealed its characteristics in both genomic structure and physiological functions that might have evolved *via* adaptation to rich milky environment and human screening for industrial application. Additional analysis for the evolutionary relationships among the genomes of the three *Lb. bulgaricus* species as well as other LAB strains indicated that strain 2038 is closer to their common ancestor than the other collection strains that might result from the strict strain maintenance process of dairy industry.

## Materials and Methods

### Genome sequencing and annotation

The *Lb. bulgaricus* 2038 genome sequence was determined by using a whole genome shotgun sequencing strategy and PCR-based gap-filling approach [6]. Two shotgun libraries were constructed, one using pUC18 as vector that was sequenced to 12.6-fold genome coverage, and the other using low-copy number vector pSMART-LCKan (Lucigen) as vector that was sequenced to 6.4-fold genome coverage. Sequencing was performed with 3730 DNA Analyzer (Applied Biosystems). After assembly by Phrap (http://www.phrap.org), 106 contigs were obtained with total size of 1.79Mb. Then PCR reactions were performed to fill the gaps Final sequence refinement was achieved by re-sequencing regions with low coverage and poor sequencing quality. Finally a single circular genome of 1,872,907 bp was obtained. Putative protein coding sequences (ORFs) were identified by Glimmer3 [7]. Functional annotation of CDSs was performed through BLASTP searches against GenBank's non-redundant (nr) protein database, followed by manual inspection. Protein domain prediction and COG [8] assignment were performed by RPS-BLAST using NCBI CDD library which integrates PFAM [9], SMART and COG. Motifs were detected by using ScanProsite [10]. Functional categories were classified according to Riley rules [11], through analyzing protein homologs and keywords of protein names. The alignment of whole genomes was performed using Mummer (http://mummer.sourceforge.net/manual/). The evolutionary rate analysis, including non-synonymous and synonymous rate, was performed using PAML [12], based on the theory of Yang Z, and Nielsen R [13].

All homologous genes in *Lb. bulgaricus* genomes (*Lb. bulgaricus* 2038, ATCC 11842, and ATCC BAA-365) were identified by BLAST. The standard for ensuring homologous genes was selected according to the method [14].

### Pathway mapping, enzyme identification and protein localization

We collected amino acid synthesis related pathway information from given pathways in Kyoto Encyclopedia of Genes and Genomes (KEGG) [15]. Through a BLAST search, genes of *Lb. bulgaricus* 2038 were mapped to EC numbers extracted from the genome annotations and manually curated. The identification of potential gene function was done by manually comparing the domains of genes from the prediction results with known enzyme domains. The presence and location of signal peptide cleavage sites was predicted by SignalP3.0 with hidden Markov models [16], transmembrane topologies were predicted by ConPred II [17], the lipoproteins were predicted using LipoP1.0 [18], and PSORTb v.2.0 [19] were used to help determine the subcellular localization of all proteins.

### Other microorganism genomes

All 16 genomes of the organisms involved in this article are derived from the NCBI (http://www.ncbi.nlm.nih.gov/): *Lactobacillus delbrueckii* subsp. *bulgaricus ATCC 11842* (NC_008054), *Lactobacillus delbrueckii* subsp. *bulgaricus ATCC BAA-365* (NC_008529), *Lactobacillus acidophilus NCFM* (NC_006814), *Lactobacillus johnsonii NCC 533* (NC_005362), *Lactobacillus sakei* subsp. *sakei 23K* (NC_007576), *Lactobacillus plantarum WCFS1* (NC_004567), *Lactobacillus salivarius* subsp. *salivarius UCC118* (NC_007929), *Lactobacillus brevis ATCC 367* (NC_008497), *Lactobacillus casei ATCC 334* (NC_008526), *Lactobacillus gasseri ATCC 33323* (NC_008530), *S. thermophilus CNRZ1066* (NC_006449), *S. thermophilus LMD-9* (NC_008532), *S. thermophilus LMG 18311* (NC_006448), *Lactococcus lactis* subsp. *lactis Il1403* (NC_002662), *Lactococcus lactis* subsp. *cremoris SK11* (NC_008527), *Lactococcus lactis* subsp. *cremoris MG1363* (NC_009004). We collected the sequence data and annotation information from the NCBI and KEGG websites.

### Phylogenetic tree construction

The strategy used for construction of phylogenetic tree has been reported previously [20]. We collected highly conservative 16S rRNAs from all genomes, aligned them by CLUSTW, and the tree was built in MEGA3 using NJ method [21] and ML method [22]. The strategy for constructing the phylogenetic tree based on genome context was a newly developed system in evolutionary research [23]. Three methods including the phylogenetic profiles method, the gene neighbors method and the gene fusion method were adapted to construct the genome context networks [20]. We used 99 species as reference species to construct the gene context networks of 17 strains. And based on the pair-wise similarity of these 17 gene context networks, we construct the phylogeny. The homologous genes within all ten strains were identified through gapped BLASTP [24], with default settings. Subsequently we aligned the networks and detected distances between species. A phylogenetic tree based on this information was built in TREEVIEW.

### Accession number

The whole genome sequence of *Lb.bulgaricus* 2038 and its annotations have been deposited in GenBank under the accession number CP000156.

## Results

### Genomic characteristics of *Lb. bulgaricus* 2038 and its intra-species pan-genomic analysis

**Figure 1:**
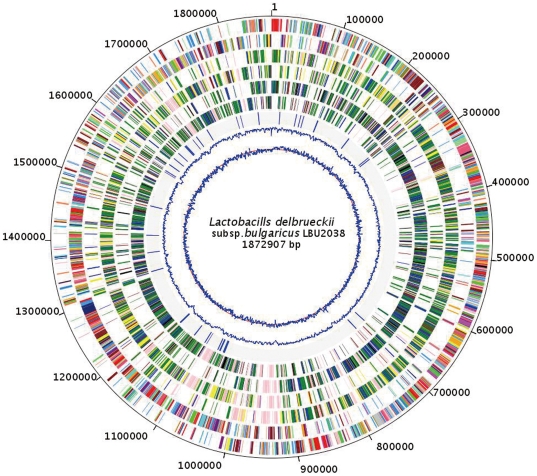
Chromosome Atlas of the chromosome of *Lactobacillus delbrueckii* subsp. *Bulgaricus* 2038. Each concentric circle, number from outermost circle to innermost circle, represents genomic data for *Lactobacillus delbrueckii* subsp. *Bulgaricus* strain LBU2038 and comparison with ACCT11842 and ACCT BAA-365. First and second circles show predicted coding sequences (ORFs) on the plus and minus strands, respectively, colored by functional role categories according to COG: [J]: “Translation, ribosomal structure and biogenesis” = salmon; [A]: “RNA processing and modification” = light blue; [K]: “Transcription” = light green; [L]: “Replication, recombination and repair” = red; [B]: “Chromatin structure and dynamics” = brown; [D]: “Cell cycle control, cell division, chromosome partitioning” = yellow; [Y]: “Nuclear structure” = green; [V]: “Defense mechanisms” = purple; [T]: “Signal transduction mechanisms” = pink; [M]: “Cell wall/membrane/envelope biogenesis” = orange; [N]: “Cell motility” = blue; [Z]: “Cytoskeleton” = grey; [W]: “Extracellular structures” = sea green; [U]: “Intracellular trafficking, secretion, and vesicular transport” = black; [O]: “Posttranslational modification, protein turnover, chaperones” = aqua; [C]: “Energy production and conversion” = fuchsia; [G]: “Carbohydrate transport and metabolism” = lime; [E]: “Amino acid transport and metabolism” = maroon; [F]: “Nucleotide transport and metabolism” = navy; [H]: “Coenzyme transport and metabolism” = olive; [I]: “Lipid transport and metabolism” = silver; [P]: “Inorganic ion transport and metabolism” = lime green; [Q]: “Secondary metabolites biosynthesis, transport and catabolism” = cadet blue; [R]: “General function prediction only” = coral; [S]: “Function unknown” = dodger blue. Third and forth circles represent the conservation of the ORFs sequences compared to ACCT11842 on the plus and minus strands, respectively, colored by conservation levels: gain gene = pink; INDEL gene = yellow; non-synonymous SNP gene = green; synonymous SNP gene = blue; equal gene = dark red. Fifth and Sixth circle display the sequence comparison of each ORF to ACCT BAA-365 on the plus and minus strands, with same colors with third and forth circles for conservation levels. Seventh circle show the IS sequences. Eighth circle present the GC content of the chromosome. Ninth circle displays the GC skew of the chromosome (red circle line represent the value of zero of GC skew).

The primary features of *Lb. bulgaricus* 2038 genome are presented in **Figure 1**. *Lb. bulgaricus* 2038 contains a single, circular chromosome of 1,872,907 bp, with an average GC content of 49.68%. We detected 1,790 CDSs in the genome, with an average length of 859bp. The average GC and GC3 (GC at codon position 3) content in CDSs are 51.59% and 64.73%, respectively (**Table 1**). Among the CDSs, 1,524 proteins can be assigned to COG families [8]. Biological functions could be defined to 1,224 (68.4%) of the predicted proteins, while the other 413 CDSs (23.1%) are homologous to conserved proteins of unknown function in other organisms. The remaining 153 hypothetical proteins (8.5%) have no match to any known proteins in the databases. At least 129 multigene (paralog) families were identified, containing 327 predicted proteins (**Table S1** in **File S2**). Altogether, the CDSs and stable RNA genes represent 84% and 2.75% of the genome, respectively.

**Table 1:** Basic information of seventeen microorganisms' genome.

Lactobacillus delbrueckii subsp. Bulgaricus	accession	genome size (bp)	GC_bp	intact ORFs	truncated ORFs	all genes	ORFLen	ORFLen Avg	CG content	CG bp in gene	CG content in gene	GC3 content %
*LBU_2038*	CP000156	1,872,907	930,460	1,661	129	1,790	1,537,377	859	49.68%	793116	51.59%	64.73%
*ATCC 11842*	NC_008054	1,864,998	927,259	1,562	270	1,832	1,370,460	877	49.72%	707387	51.62%	64.86%
*ATCC BAA-365*	NC_008529	1,856,951	922,651	1,647	74	1,721	1,432,908	833	49.69%	737965	51.50%	64.69%

All genomes in the table were obtained from the NCBI database, except sequenced *Lb. bulgaricus* 2038. GC3 reflects the GC content at the third site of each codon.

**Figure 2:**
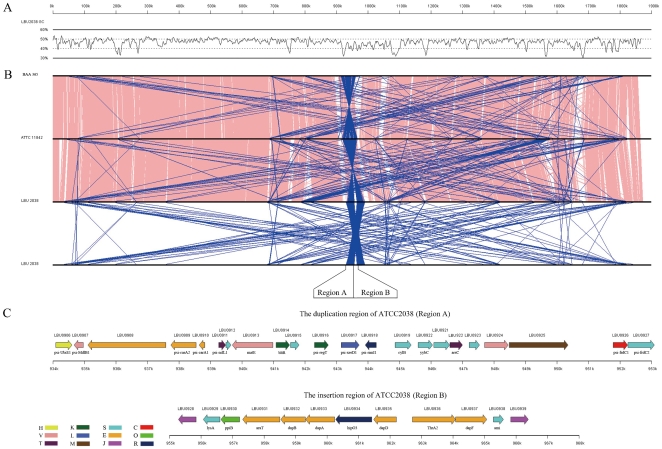
GC scan and the duplication regions of *Lb. bulgaricus* 2038 genome. (**A**) **The GC content of** ***Lb. bulgaricus*** **2038 genome (30% to 60%).** It was scanned with a window size of 5000 bp and step of 1000 bp. (**B**) **Alignment result of genomes between** ***Lb. bulgaricus*** **2038, ATCC 111842 and ATCC BAA-365 by Mummer.** Pink: same direction; Blue: reverse direction. The characteristic duplication regions (A and B) flanking the predicted replication terminus are specially labeled. (**C**) **Genes existed in the insertion and duplication regions (950k bp to 968k bp) of** ***Lb. bulgaricus*** **2038.** Their corresponding COG families are labeled by colors with letter specifications. [C]: Energy production and conversion; [E]: Amino acid transport and metabolism; [H]: Coenzyme transport and metabolism; [J]: Translation, ribosomal structure and biogenesis; [K]: Transcription; [L]: Replication, recombination and repair; [M]: Cell wall/membrane/envelope biogenesis; [O]: Posttranslational modification, protein turnover, chaperones; [R]: General function prediction only; [S]: Function unknown; [T]: Signal transduction mechanisms; [V]: Defense mechanisms.

**Figure 3:**
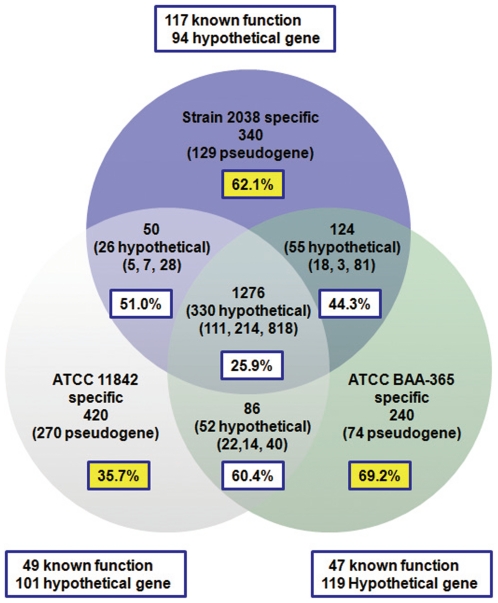
Homologous genes in the strains of *Lb. bulgaricus*. Orthologous genes existed in each pair of strains and in all three strains of *Lb. bulgaricus* are counted and shown in corresponding sectors. Three types of orthologous genes were counted and illustrated in the parentheses from left to right: genes with identical DNA sequence, genes with synonymous variations, and genes with amino acid variations. Each orthologous gene pair was identified based on the best Blast hit (BBH) approach. The numbers in the white square frames represent the percentage value of hypothetical genes in each category. The numbers in the non-overlap segments of Venn Diagram represent the number of non-orthologous genes specific to each of the three strains, consisting of two types, unique vs. duplicated. The numbers in the yellow square frames represent the percentage value of pseudogenes in each strain. The numbers in the outside frames of the diagram represent the numbers of the annotated (known function) genes and hypothetical genes in the part of non-orthologous genes specific to each strain.

The genome size of *Lb. bulgaricus* 2038 is slightly larger han that of the other two sequenced collection strains of *Lb. bulgaricus* (1,864,998 bp for *Lb. bulgaricus* ATCC 11842 and 1,856,951 bp for ATCC BAA-365). All of these strains have two duplicated segments (25kb in length in strain 2038), while there is an unique central region (8.5kb) between the duplication regions in *Lb. bulgaricus* 2038 (**Figure 2**). The intra-species pan-genome [25] of the three *Lb. bulgaricus* consists 1276 ‘core’ genes (**Figure 3**). There were 211 ‘strain-specific’ genes identified in *Lb. bulgaricus* 2038 (**Table S2** in **File S2**), more than those in either strain ATCC 11842 (150) or strain ATCC BAA-365 (166).

The relative evolutionary rates were analyzed by comparing the rates of non-synonymous (dN) and the rate of synonymous substitution (dS) of genes among the *Lb. bulgaricus* pan-genomes at the level of either whole genome or COG categories (**Table S3** and **Table S4** in **File S2**). The evolutionary rates for the genes in the categories of Coenzyme Transport and Metabolism [H] and Intracellular Trafficking, Secretion, and Vesicular Transport [U] are significantly higher in *Lb. bulgaricus* 2038 than in the other two strains. In addition, genes in the category of Methionine Metabolism has the value of dN/dS greater than 1 (**Table S5** in **File S2**), attributed by the high rate of non-synonymous substitution of *metK* (LBU1348), encoding methionine adenosyltransferase [EC 2.5.1.6] that catalyzes the formation of S-adenosylmethionine (SAM) from methionine. As the other genes in SAM cycle remain conserved (**Table S4** in **File S2**), probable positive selection of *metK* gene in *Lb. bulgaricus* 2038, and in turn, for SAM synthesis capacity, and transmethylation reactions, is implicated.

### Strain-specific genetic features of *Lb. bulgaricus* 2038 implicating its industrial application related evolving process

The *Lb. bulgaricus* 2038 genome is larger than that of the other two collection strains, indicating that along with SNPs, presence of extra genes are major genetic characteristics to endow its specific features that is probably related to its industrial applications.

#### Unique lysine synthesis capability

Genomic analysis revealed that *Lb. bulgaricus* 2038 is the only strain capable of *de novo* synthesizing lysine among the three sequenced *Lb. bulgaricus* strains. All of the 7 genes for a complete lysine biosynthetic pathway (**Figure S1** in **File S1**) (*LBU0931*-*LBU0937*) were strain-specific to *Lb. bulgaricus* 2038 and located in the 8.5kb central region between the two duplicated segments (**Figure 2C**). It is interesting that, besides *Lb. bulgaricus* 2038, the complete pathway for lysine biosynthesis can also be found in the genomes of *Lb. salivarius*, *Lb. acidophilus*, *Lb. plantarum* and *Lb. casei*. Among them, only *Lb. salivarius* possess tetrahydrodipicolinate N-acetyltransferase [EC 2.3.1.89] similar to that of *Lb. bulgaricus* 2038, while the others utilize 2,3,4,5-tetrahydropyridine-2-carboxylate N-succinyltransferase [EC 2.3.1.117] instead. Although it is reported that *Lactococcus lactis* could synthesize lysine [26], *dapF* encoding diaminopimelate epimerase [EC 5.1.1.7] is yet to be identified in its genome. Besides, gene loss events were found in other *Lactobacillus* species that had made them lose the capability of *de novo* lysine synthesis (**Table 2**). It seems that in *Lb. helveticus* and *Lb. fermentum*, they merely lack the *araT* encoded aromatic amino acid aminotransferase [EC 2.6.1.57], while the genomes of *Lb. sakei* 23K and *Lb. brevis* ATCC 367 lose all the genes involved in this pathway.

**Table 2:** Enzymes in the pathway of Lysine biosynthesis.

L-Aspartate→L-Lysine biosynthesis									
Gene name	lysC	asd	dapA	dapB	dapD	araT	hipO3	dapF	lysA
EC	2.7.2.4	1.2.1.11	4.2.1.52	1.3.1.26	2.3.1.892.3.1.117	2.6.1.57	3.5.1.47	5.1.1.7	4.1.1.20
*Lb. bulgaricus 2038,* *Lb. salivarius,* *Lb. acidophilus,* *Lb. casei,* *Lb. plantarum,* *Lb. reuteri*	*	*	*	*	*	*	*	*	*
*Lc. Lactis,* *St. thermophilus*	*	*	*	*	*	*	*		*
*Lb. fermentum*,*Lb. helveticus*	*	*	*	*	*		*	*	*
*Lb. bulgaricus ATCC BAA-365*	*	*				*			*
*Lb. bulgaricus ATCC 11842,* *Lb. gasseri*	*	*				*			
*Lb. johnsonii*						*			
*Lb. sakei,* *Lb. brevis*									

Enzymes in the biosynthesis of L-Lysine from L-Aspartate are listed in the table.

*denotes that the enzyme is existing in the species.

#### Enzymes for converting aspartate into carbon-skeleton intermediates

A phosphoenolpyruvate carboxykinase encoding gene (*PckA*, *LBU0686*) was identified in *Lb. bulgaricus* 2038, while the counterpart orthologs in either *Lb. bulgaricus* ATCC11842 or *Lb. bulgaricus* BAA365 were found to be pseudogenes caused by INDELs. The PckA enzyme catalyzed the synthesis of phosphoenolpyruvate (PEP) from oxaloacetate (OAA). Together with two aspartate transaminases [EC 2.6.1.1], encoded by *LBU0363* and *LBU1079*, catalyzing the reaction to convert aspartate into OAA, PckA endowed *Lb. bulgaricus* 2038 with an ability to bring aspartate into the carbon intermediate cycle. As the key enzyme of gluconeogenesis - fructose 1,6-bisphosphatase - was not encoded in *Lb. bulgaricus*, PEP would be finally transformed into either lactate fermentation-based catabolism or into acetyl-CoA for generating metabolic intermediates. Although *Lb. bulgaricus* grew in an environment rich in both protein and lactose, the unique ability of using aspartate for carbon metabolism might be important for its fast growth, particularly, when the ratio of carbon *vs.* nitrogen in the medium is suboptimal during milk fermentation (personal communication, Meiji Dairies Corporation).

#### Formate production

It has been known and supported via comparative genomic analysis of *S. thermophilus* and *Lb. bulgaricus*, that the complentary formate supply from the co-cultivated *S. thermophilus* is essential for successful industrial application of *Lb. bulgaricus* [4]. However, there is a probable pathway converting GTP to formate identified in *Lb. bulgaricus* 2038. We found that gene *LBU1742* gene encoded a GTP cyclohydrolase II [EC:3.5.4.25], which catalyzes the first committed reaction in the biosynthesis of riboflavin, may convert GTP into a mixture of pyrophosphate, formate, and 2,5-diamino-6-ribosylamino-4 (3*H*)-pyrimidinone 5′-phosphate [27]. Because there is no orthologs found in either *Lb. bulgaricus* ATCC11842 or *Lb. bulgaricus* BAA365, the ability of releasing formate from GTP is specific for *Lb.bulgaricus* 2038. This kind of formate production might satisfy the requirement of *Lb. bulgaricus* 2038 when GTP or its precursor are supplied (*e.g.*, in the rich medium of seeding culture), and thus endow its growth advantage comparing to other *Lb. bulgaricus* strains.

#### Special exopolysaccharides (EPS) synthesis

Two neighboring *eps* clusters with significant differences in gene contents were identified in the genomes of the three *Lb. bulgaricus* strains (**Table S6** in **File S2**). In *Lb. bulgaricus* 2038, these two clusters have lengths of 16-kb (*LBU1630*-*LBU1618*) and 12-kb (*LBU1598*-*LBU1588*), respectively (**Table S7** in **File S2**). Compared to *Lb. bulgaricus* ATCC11842 and *Lb. bulgaricus* BAA365, the 16-kb *eps* cluster of *Lb. bulgaricus* 2038 has 8 unique genes, including 5 genes (*epsF*, *epsG*, *cpoA2*, *epsJ*, *epsM*) encoding glycosyl transferases (GTFs) (**Figure S2A** in **File S1**). This *eps* cluster is highly homologous with an known 18-kb *eps* cluster of *Lb. bulgaricus Lfi5* [28] (**Figure S3** in **File S1**), with the only difference that *epsH* and *epsI* of *Lfi5* which encode galactosyltransferases are displaced by *LBU1623* in strain 2038 encoding a putative lipopolysaccharide N-acetylglucosaminyltransferase. The 12-kb *eps* cluster of 2038 also showed significant differences against *Lb. bulgaricus* ATCC11842 and *Lb. bulgaricus* BAA365 with respect to gain and loss of some GTFs encoding genes (**Figure S2B** in **File S1**). Therefore, the EPS repeat unit produced by either the 16-kb or the 12-kb *eps* clusters of 2038 must be different from that of ATCC11842 and BAA365, which should account for the difference of texture or mouse-feeling of the yogurt thus produced.

#### Type II restriction-modification (RM) systems

*Lb. bulgaricus* 2038 encodes two complete type II RM systems (*LBU0994*, *LBU0995*; *LBU1698*, *LBU1699*) and two inactive type I RM systems (*LBU0895*; *LBU1045*, *LBU1046*, *LBU1048*, *LBU1049*, *LBU1050*) (**Table S8** in **File S2**). In contrast, the *Lb. bulgaricus* ATCC 11842 contains a complete type I RM system (*Ldb1051*, *Ldb1052*, *Ldb1053* and *Ldb1055*) and an inactive type III system [4], while the *Lb. bulgaricus* ATCC BAA-365 contains only one type II RM system. Only moderate or even little orthologs were shown among genes encoding the RM systems in the three strains (**Table S8** in **File S2**). The truncated *LBU0895* is highly similar to *Ldb1051* of type I RM system in *Lb. bulgaricus* ATCC 11842, reflecting a similar RM system once existed in *Lb. bulgaricus* 2038 but now degenerated. Although one of the two type II RM systems (*LBU0994*, *LBU0995*) in *Lb. bulgaricus* 2038 is not found in the other two strains, the *LBU1698*/*LBU1699* system does have homologous genes in *Lb. bulgaricus* ATCC BAA-365.

Despite the highly diversified RM systems found in the three strains, the DNA sequences in the regions around the type I RM system of *Lb. bulgaricus* ATCC 11842 and those around the type II RM systems of *Lb. bulgaricus* 2038 are conserved in all three *Lb. bulgaricus* strains (**Figure S4** in **File S1**). These conserved adjacent regions may imply that the RM systems in the three stains were once similar, but eventually evolved in different directions.

#### Extra mismatch repair genes

MutS and MutL dependent long-patch mismatch repair system was responsible for correcting errors during DNA replication [29]. Three *mutS* genes (*LBU0078*, *LBU1364* and *LBU1377*) and one *mutL* gene (*LBU1376*) was identified in *Lb. bulgaricus* 2038, but only two *mutS* genes were identified in the other two *Lb. bulgaricus* strains (ATCC11842 and BAA365). The *Lb. bulgaricus* 2038 specific *mutS* gene (*LBU0078*) might offer the strain a stronger capability to maintain its DNA fidelity in replication.

### Genomic characteristics shared by *Lb. bulgaricus* strains associated with industrial features

Besides the specific features of *Lb. bulgaricus* 2038, we also revealed some industrial features associated with genes shared by all three of the *Lb. bulgaricus* strains. These features should be pre-selected in the *Lb. bulgaricus* population along evolution before their usage in industry; meanwhile most of them were retained in the long-term evolutionary selection because of their contribution to its application in yogurt production.

#### Nitrogen metabolism-protocooperation with *Streptococcus thermophilus*

Only four amino acids (aspartate, asparagine, lysine and threonine) could be *de novo* synthesized by *Lb. bulgaricus* 2038 whereas only three (except lysine) for the other two collected strains, Therefore, *Lb. bulgaricus* has to obtain exogenous amino acids or peptides for growth. There are at least 49 genes encoding putative proteases or peptidases in the *Lb.bulgaricus* 2038 genome, including one cell wall-anchored protease (PrtB, LBU1015) and two extracellular peptidases (LBU1040 and LBU1705). These extracellular protease/peptidases were essential for efficient utilization of environmental proteins of *Lb. bulgaricus* 2038 and co-cultured *S. thermophilus*, which was known to lack cell wall-anchored protease [30]. Among the peptidases, LBU1040 (extracellular Peptidase M23) and LBU0520 (cytoplasmic D-aminopeptidase) has no ortholog in *Lb. bulgaricus* ATCC BAA-365, and there's no ortholog of LBU1705 (extracellular dipeptidase), LBU0521 (cytoplasmic L-aminopeptidase) or LBU0898 (cytoplasmic metal-dependent amidase/aminoacylase/carboxypeptidase) in *Lb. bulgaricus* ATCC 11842. In contrast, all the proteases/peptidases of *Lb. bulgaricus* ATCC 11842 and *Lb. bulgaricus* ATCC BAA-365 have their orthologs found in *Lb. bulgaricus* 2038. The vast number of protease/peptidase was complimented by 10 ATP binding cassette (ABC)-type transport systems for peptides and amino acids as well as six amino acid permeases. There is no ortholog for amino acid permease LBU1506 and ABC-type amino acid transport system LBU0240/LBU0241/LBU0242 in *Lb. bulgaricus* ATCC 11842 either.

#### D-lactic acid production

*Lb. bulgaricus* produces D-lactic acid from four sugars (lactose, glucose, fructose and mannose) *via* the Embden-Meyerhof-Parnas (EMP) pathway and is incapable of fermenting pentoses. Two isomeric forms of lactate, D(−) and L(+), may be formed *via* reduction of pyruvate by distinct stereospecific NAD-dependent lactate dehydrogenases (D-LDH or L-LDH). Although there are two genes encoding L-LDH (*ldhL1*, *LBU0059*; *ldhL2*, *LBU0084*), they are unlikely to be expressed at significant levels, as indicated by their low codon adaptation indices (0.305 and 0.225, respectively; Table S3 in **File S2**) [31]. Conversely, among the 3 genes encoding D-LDH, *ldhD1* (*LBU0066*) was scored as nearly the highest expressed gene in the genome (CAI = 0.575), while *ldhD2* (*LBU0860*) and *ldhD3* (*LBU1637*) seem to be moderately expressed (CAI values of 0.369 and 0.320, respectively). This may account for the fact that in *Lb. bulgaricus*, more than 90% of the pyruvate is converted to D-lactate [32].

#### Lower flavor compounds production capability favors artificial adjustment after fermentation

Pyruvate is the precursor of many short-chain flavor compounds [33]. However, in the genomes of *Lb. bulgaricus* strains, pyruvate dissipating enzymes converting pyruvate to acetaldehyde, acetoin, diacetyl and acetone are lost (**Figure S5** in **File S1**). Though acetaldehyde and acetic acid can be synthesized through acetyl-phosphate (Ac-P), the conversion from pyruvate to Ac-P needs oxygen, which is rare under the fermentation condition.

Amino acids are major precursor for flavor compounds [34]. In *Lb. bulgaricus* 2038, *LBU1116* encoded aminotransferase could transfer branched-chain amino acids into corresponding α-keto acids, which are known to have cheesy flavours [35]. *LBU1014* encoded cystathionine β-lyase could convert methionine to methanethiol with a very low efficiency [36], which is a flavor compound in many foods and can be further transformed into thioesters. Except these two genes, many other known flavor associated genes are absent in *Lb. bulgaricus* genome, like glutamate dehydrogenase (GDH) and threonine aldolase.

#### Stress tolerance — essential for industrial fermentation

A thioredoxin system including two thioredoxin reductases (LBU0516, LBU1349, TrxB) and two thioredoxins (LBU1306, LBU1363) was assumed playing an important role in *Lb. bulgaricus* oxygen tolerance. Other antioxidant enzymes found in the genome include peptide methionine sulfoxide reductase (LBU0568, LBU1612) and LBU0652, which showed some homologous to glutathione-disulfide reductase [EC 1.8.1.7]. In addition, a functional RecA protein (encoded by *LBU0503*) is likely to play an important role in repairing oxidative DNA damage.

One operon encoding eight genes (*LBU0598*-*LBU0605*) of the F0F1-ATPase system was predicted in the *Lb. bulgaricus* 2038 genome, serving as a major regulator of intracellular pH by extruding protons in expense of ATP [37]. Meanwhile three cation transport ATPases (LBU0271, LBU0681, LBU1113) and three Na+-H+ antiporters (LBU0181, LBU1525, LBU1758) help maintain the intracellular pH equilibrium through the exchange of cation for H^+^. Two ornithine decarboxylases (LBU0458, LBU1505) catalyze the transformation from ornithine to putrescine and consume a proton for each reaction, which will increase the intracellular pH [38]. Some known genes associated with cell membrane biogenesis and stability at low pH, like *dlt* operon (*LBU1749*-*LBU1752*) and *ffh* gene (*LBU1180*) are also found in *Lb. bulgaricus* 2038 genome. In addition, extracellular housekeeping protease HtrA (LBU0100) may degrade aberrant proteins synthesized under stress conditions [39]. Chaperone GroES (LBU1379), GroEL (LBU1378) and DnaK (LBU1124) are known to be related to acid response in *Lb. bulgaricus* [40], perhaps playing protective roles for protein stability under acidic conditions.

### Genome relationship network construction-based phylogenetic analysis

**Figure 4:**
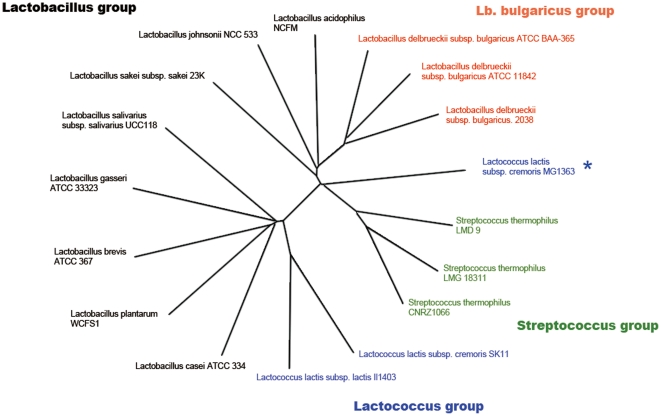
Phylogenetic trees for genome wide comparative analysis. Phylogenetic trees of seventeen bacteria based on the basis of the alignments of gene relationship networks derived from genome context information, with all branches supported at >70% bootstrap values. All notes from gene networks are homologous genes in these microorganisms, and the branches in the gene networks are constructed using three kinds of relationships: predicted by phylogenetic profiles method, gene neighbors method and gene fusions method.

To investigate the phylogenetic relationship of LAB species [5], [41], the phylogenetic tree including genomes of 17 strains belonging to 12 species of LAB particularly including the three strains (2038 and the other two ATCC strains) of subspecies of *Lb. bulgaricus* (see in *Methods*) was constructed based on the similarity of 16S ribosomal RNAs (**Figure S7 and Figure S8** in **File S1**). As expected, it is the same as the one previously reported, where the strains of the same subspecies are generally indistinguishable. However, in a genome context network (GCN, hereafter) based phylogenetic tree (**Figure 4**), potential evolutionary relationship of the LAB strains can be illustrated among strains of the same species such as that for *Lb. bulgaricus* (three strains for this study), *Lactococcus lactis* subsp. cremoris (two strains) and *S. thermophilus* (three strains). The GCN-based phylogenetic tree illustrated that, (i) at the species level, the three groups are no longer clustered closely as in the 16S rRNA-based tree; (ii) subtle differences were observed in the relationships of subgroups. Particularly, in the *Lb. bulgaricus* subfamily, ATCC 11842 and ATCC BAA-365 are much closer to each other than to *Lb. bulgaricus* 2038 in the GCN tree.

These slight differences of strains within one species cannot be detected in the 16S rRNA-based tree because they have almost identical 16S rRNA sequences. This overall view for the variation of the structure of gene network among different strains of *Lb. bulgaricus* echoed well with previous comparative genomic analysis based on the information of gene lost and gains. Combining the internal branching topology among the strains of *Lb. bulgaricus* and the other genomic evidence mentioned above, we speculate that *Lb. bulgaricus* 2038 is closer to the common ancestral strain of *Lb. bulgaricus* than the other two, which might have lost more metabolic pathways or evolved more dramatically from their ancestral genome.

## Discussion

In this article, we reported and analyzed, for the first time, the complete genome of an industrial *Lb. bulgaricus* strain 2038 that has been used decades for yogurt fermentation. We compared the genomic information of *Lb. bulgaricus* 2038 within either its taxonomic subspecies group or the related industrial LAB group, and revealed the genetic basis for the characteristics probably related with industrial yogurt production of this economically important strain.

The intra-species pan-genome [25] of *Lb. bulgaricus* (**Figure 3**) was studied at both structural (genomic sequence) and the functional (gene) levels. The genome of strain 2038 is structurally similar to that of the other two collection strains, with its size being slightly bigger (**Table 1**). The existence of the 8.5kb central region between the inverted duplication is the major contribution for this difference. Because there is no evidence indicating that this 8.5kb region is an extrinsic gene island, it is likely that *Lb. bulgaricus* 2038 inherits it from its ancestor, while the two closely related collection *Lb. bulgaricus* strains, ATCC 11842 and ATCC BAA-365, lost it as they evolved separately and independently.

Besides the unique capability of lysine biosynthesis, *Lb. bulgaricus* 2038 possesses four peptidases, one amino acid permease and an ABC-type amino acid transporter system that do not have orthologs in one of the other two collection strains. Therefore, it should be more efficient than the other two strains in protein utilization for both *Lb. bulgaricus* and the co-cultured *S. thermophilus*.

The presence of a *pckA* gene in strain 2038 endowed the strain the ability to convert aspartate into carbon cycles, which might be critical in media with lower C/N ratios. While the supply of formate from GTP catalyzed by GTP cyclohydrolase II might endow strain 2038 growth advantage comparing to other *Lb. bulgaricus* strains.

It is known that EPSs may reduce syneresis and improve texture, viscosity and mouth-feeling of fermented milks [42], [43]. *Lb. bulgaricus* 2038 synthesizes unique EPS different from that of ATCC 11842 and ATCC BAA365. Depending on the EPS structure of the 18-kb *eps* cluster of *Lb. bulgaricus* Lfi5, we presume that the 16-kb *eps* cluster of *Lb. bulgaricus* 2038 encode enzymes responsible for synthesizing a hexasaccharide repeating unit, composed of Galactose, Glucose, Rhamnose and N-acetylglucosamine in the ratio of 3∶1∶1∶1 (**Figure S6** in **File S1**). *Lb. bulgaricus* 2038 produced little flavor compounds since the main enzymes responsible for flavor compounds production are absent in *Lb. bulgaricus* 2038. It is actually a beneficial for artificial adjustment of yoghort flavor after fermentation. Although there are five LDH encoding genes, the highly expressed D-LDH guarantee the high efficient production of D-lactate. Meanwhile, efficient acid resistant system ensured the growth of *Lb. bulgaricus* 2038 when large amount of lactate is produced.

The distinct RM systems found in the three *Lb. bulgaricus* strains demonstrate that the RM systems among them are highly diverse, and probably have different effect on conferring resistance to phage contamination during fermentation [44]. Because RM systems impose barriers on gene transfer [45], *Lb. bulgaricus* 2038 may sustain a more stable genome structure with its two complete type II RM systems. In contrast,,*Lb. bulgaricus* ATCC 11842 and ATCC BAA-365 only have one active RM system.The genetic stability of *Lb.bulgaricus* 2038 is also maintained by its mismatch repair system, which contains one additional *mutS* gene (*LBU0078*) specific for *Lb. bulgaricus* 2038. MutS protein plays the role in mismatch DNA recognition [46], and the additional *mutS* gene may help *Lb. bulgaricus* 2038 to recognize DNA mismatch more efficiently and maintain its industrial features.

Ma and Eaton [47] observed that colonial strains capable of eliminating H_2_O_2_ could protect neighboring strains against attack by environmental H_2_O_2_. Therefore, co-cultivation with *S. thermophilus* could improve the oxygen tolerance of *Lb. bulgaricus*, since *S. thermophilus* possesses several enzymes providing H_2_O_2_ toleration, such as a manganese-containing superoxide dismutase (MnSOD), a thiol peroxidase, a Dpr protein and a H_2_O-forming NADH oxidase [30]. *S. thermophilus* is known to provide *Lb. bulgaricus* with ornithine and formate [30] while obtain putrescine from *Lb. bulgaricus* [4]. The process of ornithine transforming to putrescine promotes the intracellular pH [38], and helps *Lb. bulgaricus* deal with acidic environment.

Previous observations indicated that genes were lost from many LAB strains independently along evolution [5], [48]. The genomic analysis for *Lb. bulgaricus* 2038 further supported the notion that all *Lactobacillale* strains have undergone a general process of genome decay, which is characterized by loss of genes. However, comparing with *Lb. bulgaricus* ATCC 11842 and *Lb. bulgaricus* ATCC BAA-365, *Lb. bulgaricus* 2038 was found to retain more ancestor genes favorable for industrial yogurt production, such as acquiring essential metabolic intermediates (*e.g.*, lysine biosynthesis, converting aspartate into carbon intermediate, and abundant proteolytic systems) and maintaining chromosomal stability (*e.g.*, efficient RM system and extra *mutS* gene). We assume that these specific features could be attributed to strain 2038 being originally screened as an industrial strain, and then, some negative selection pressure under the industrial environment might slow down the process of genome decay and help to maintain these advantageous features.

## Supporting Information

File S1 **Supplementary figures.** Additional Word file contains supplementary figures from Figure S1 to Figure S8.
(DOC)

File S2 **Supplementary tables.** Additional Excel file contains supplementary tables from Table S1 to Table S8.
(XLS)
