# Supplementary material for: Complete Sequencing and Pan-Genomic Analysis of Lactobacillus delbrueckii subsp. bulgaricus Reveal Its Genetic Basis for Industrial Yogurt Production
Source: PLoS One. 2011 Jan 17;6(1):e15964. doi: 10.1371/journal.pone.0015964 (PMC3022021; doi:10.1371/journal.pone.0015964)

**Supplementary File 1 - File S1**

**Figure S1. The complete biosynthetic pathway of Lysine starting form Aspartate.** The genes involved in each step of *Lb.bulgaricus* 2038 were listed.


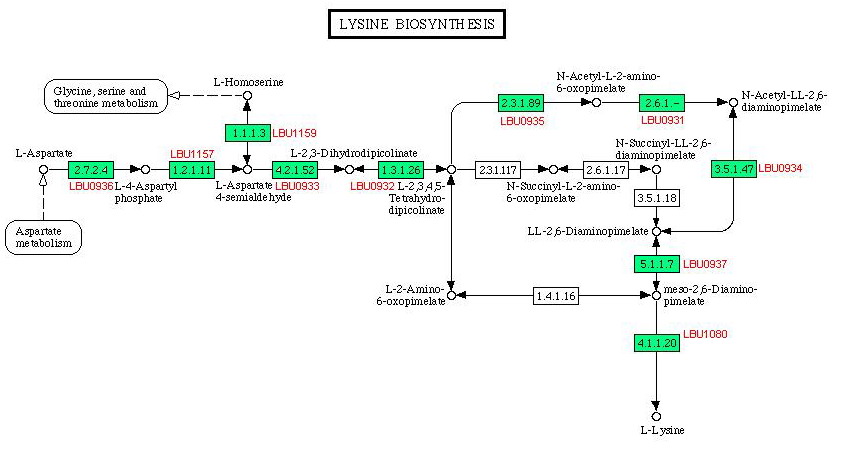


**Figure S2. Comparison of the *eps* cluster of *Lb. bulgaricus* 2038 against ATCC11842 and BAA365. Panel A.** the 16-kb *eps* cluster; **Panel B.** the 12-kb *eps* cluster.

**
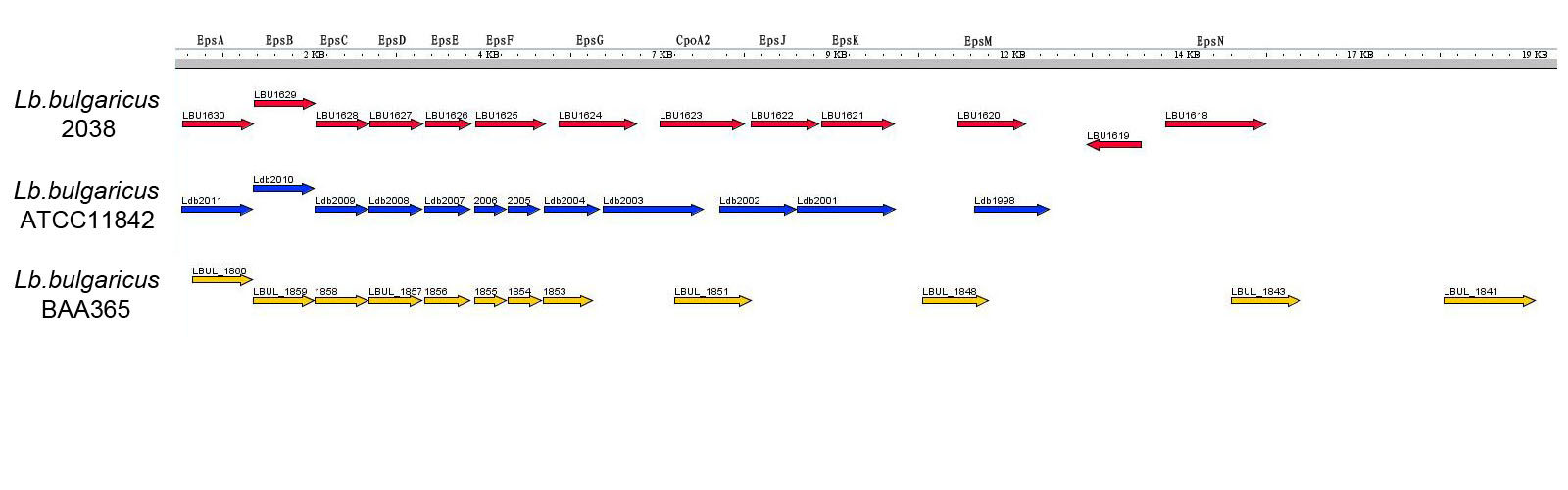
**

**
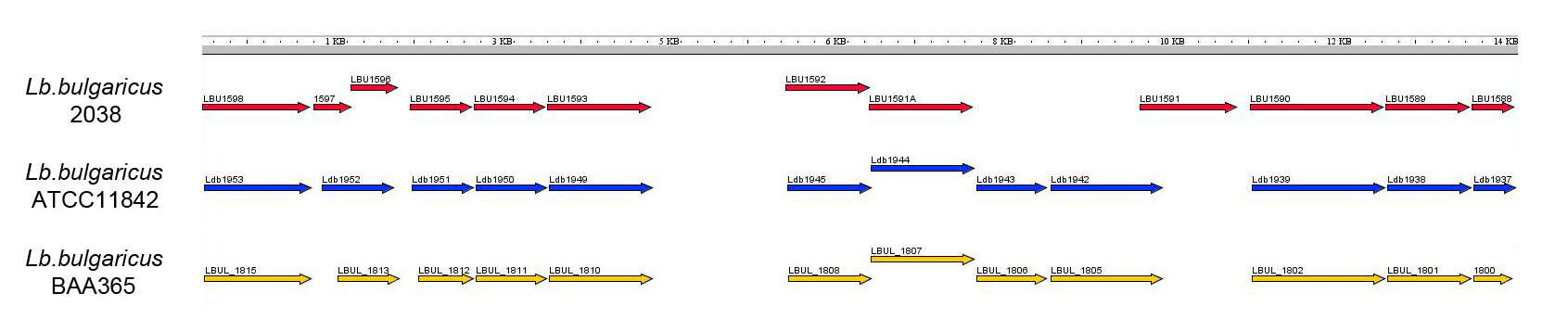
**

**Figure S3. The comparison of 16-kb *eps* cluster of *Lb. bulgaricus* 2038 and 18-kb *eps* cluster of *Lb.bulgaricus* Lfi5.** The cyan boxes represent genes involved in EPS synthesis, and the red block in the middle indicates homologous DNA regions of the two *eps* clusters.

**
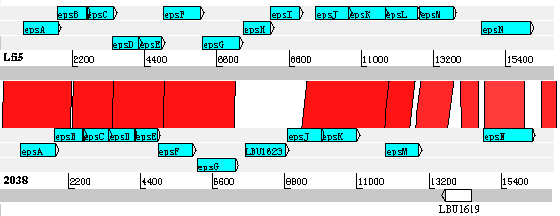
**

**Figure S4. Comparison of the regions containing RM systems in all three *Lb. bulgaricus* strains. Panel A.** Region containing type I RM system in *Lb. bulgaricus* ATCC11842 and its homologous region in *Lb. bulgaricus* 2038 and *Lb. bulgaricus* BAA365. **Panel B.** Region containing type II RM system in *Lb. bulgaricus* 2038 (*LBU0994, LBU0995*) and its homologous region in *Lb. bulgaricus* ATCC11842 and *Lb. bulgaricus* BAA365. **Panel C.** Region containing type II RM system in *Lb. bulgaricus* 2038 (*LBU1698, LBU1699*) and its homologous region in *Lb. bulgaricus* ATCC11842 and *Lb. bulgaricus* BAA365. The red blocks between two genomes represent homologous regions, and blank region represents specific DNA sequence.

**
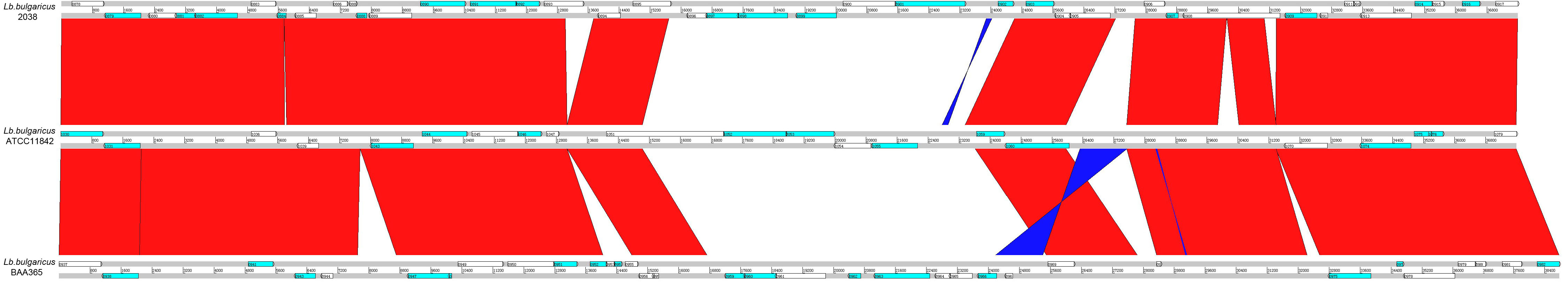

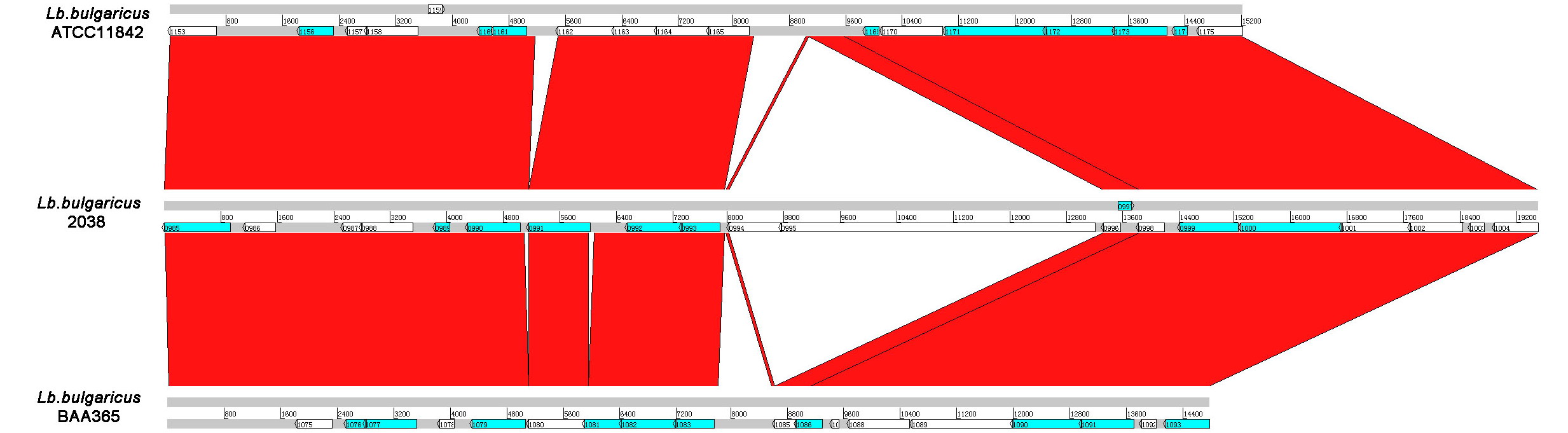

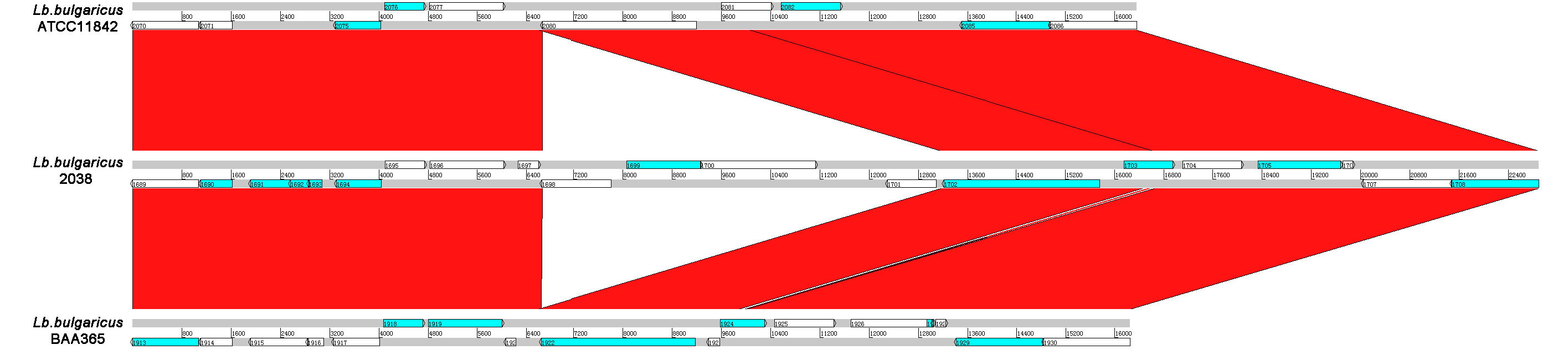
**

**Figure S5. Schematic diagram of flavor compounds production through pyruvate dissipation.** Dashed line means this step is absent in *Lb. bulgaricus* 2038, and real line means this reaction can be catalyzed by the genes of *Lb. bulgaricus* 2038.


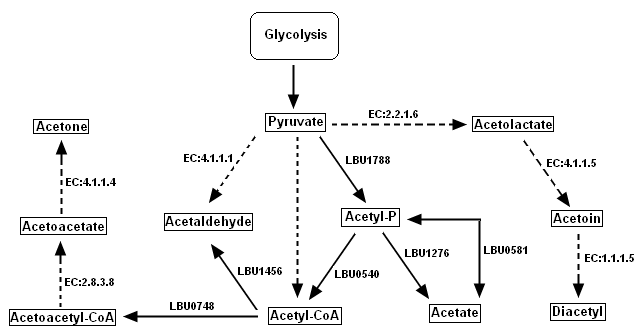


**Figure S6. Structure of the repeating unit of the EPS synthesized by *Lb. bulgaricus* 2038 and Lfi5.** The gycosyltransferase catalyzing sugar residues addition was listed adjacent to each sugar residues. The 18-kb *eps* cluster of *Lb. bulgaricus* Lfi5 encodes enzymes responsible for synthesizing a heptasaccharide repeating unit, composed of Galactose, Glucose, and Rhamnose in the ratio of 5:1:1.

**
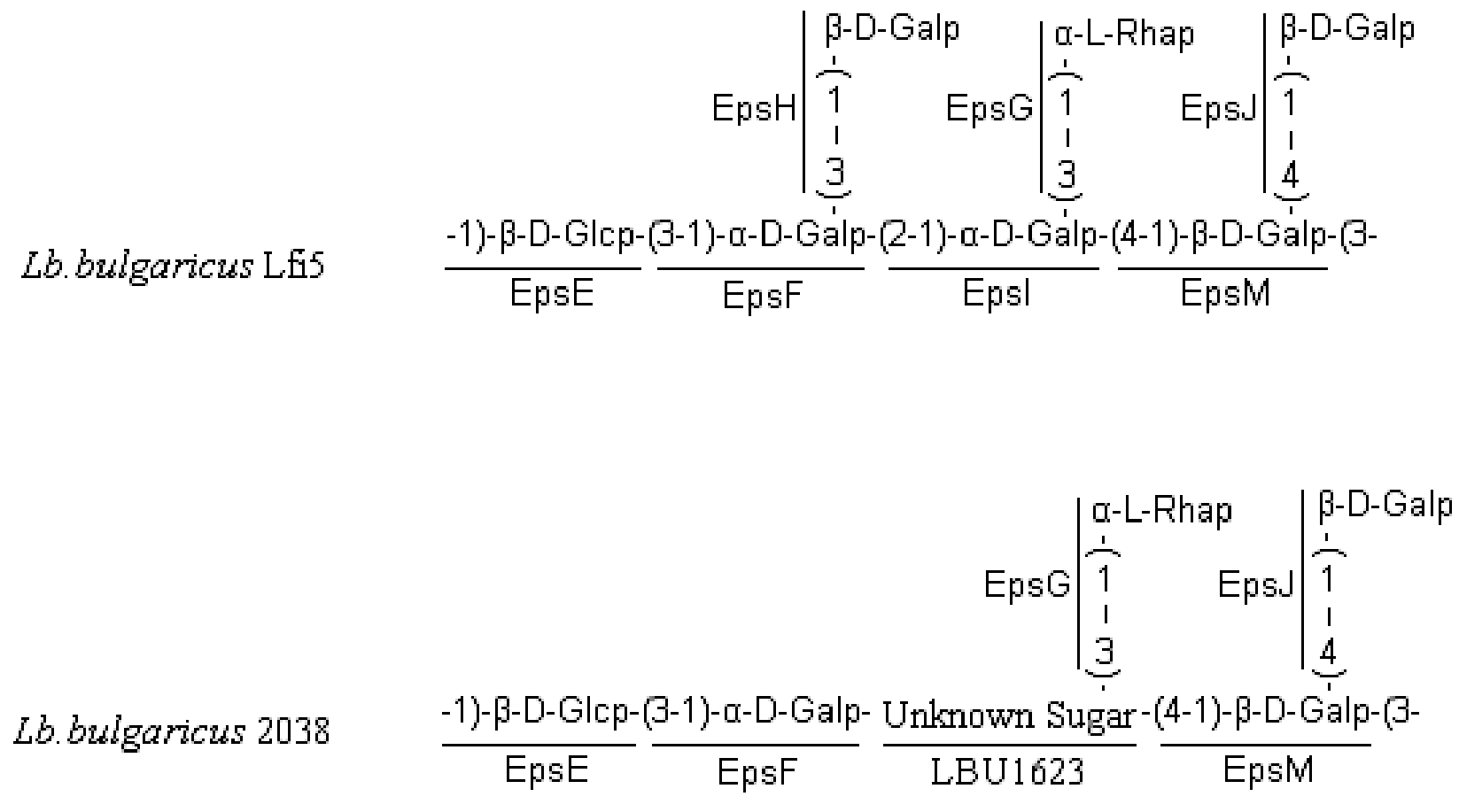
**

**Figure S7 - Phylogenetic tree (NJ) for genome wide comparative analysis.** Phylogenetic trees of seventeen bacteria based on alignments of 16S ribosomal RNA sequences using NJ method with bootstrapping.


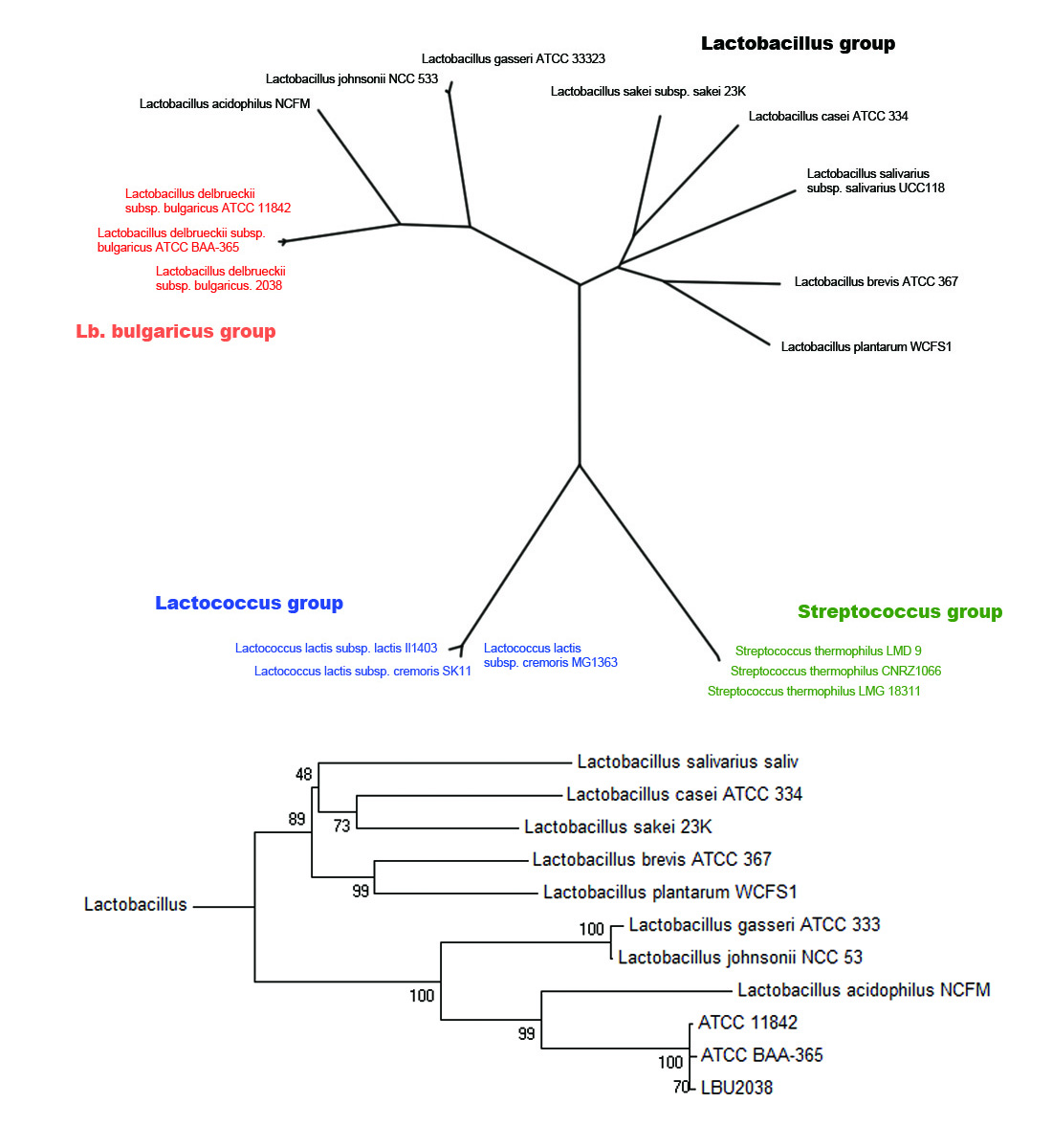


**Figure S8 - Phylogenetic tree (ML) for genome wide comparative analysis.** Phylogenetic trees of seventeen bacteria based on alignments of 16S ribosomal RNA sequences using ML method with bootstrapping.


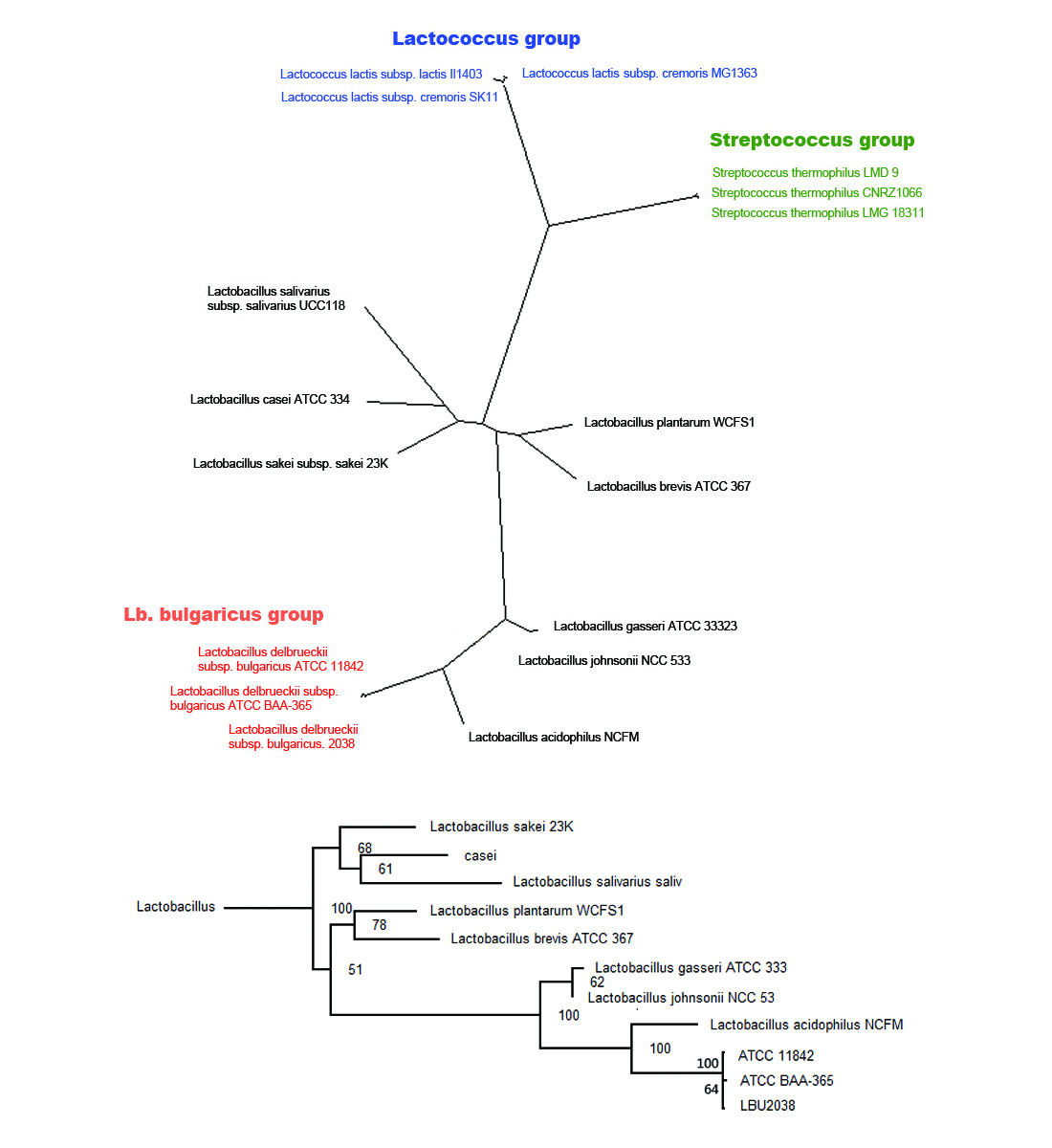

Supplement: File S1 — Supplementary figures. Additional Word file contains supplementary figures from Figure S1 to Figure S8. (DOC) [file pone.0015964.s001.doc]
